# Supplementary material for: Enhancing energy balance in wireless sensor networks through optimized minimum spanning tree
Source: PeerJ Comput Sci. 2024 Sep 26;10:e2269. doi: 10.7717/peerj-cs.2269 (PMC11623227; doi:10.7717/peerj-cs.2269)
Supplement: Supplemental Information 4 [file peerj-cs-10-2269-s004.pdf]

جامعة الملك سعود (034)
+966 11 467 56 14 هاتف
+966 11 467 42 54 فاكس

المملكة العربية السعودية
ص.ب 800 الرياض 11421
www.ksu.edu.sa

جامعة الملك سعود
King Saud University
كلية الهندسة

Date:15/08/2024
Riyadh, Saudi Arabia

To whom it may concern

This letter is to formally notify that the employment contract of Mr. Mohammed A El-Meligy with the Industrial Engineering Department, College of Engineering, King Saud University, Riyadh, Saudi Arabia, concluded on the 15th of July, 2024.

Mr. El-Meligy has served with dedication and professionalism during his tenure with the department. His contributions to our academic and research endeavors have been highly valued, and we wish him success in all future endeavors.

Please accept my sincere greetings and appreciation.

Dr Ibrahim Abdullah Almuhaidib
15/8/2024
Chirman of
Industrial Engineering Department

الرقم
التاريخ
المرفقات
